# Loss of *Ikbkap/Elp1* in mouse oocytes causes spindle disorganization, developmental defects in preimplantation embryos and impaired female fertility

**DOI:** 10.1038/s41598-019-55090-1

**Published:** 2019-12-11

**Authors:** Kuo-Tai Yang, Azusa Inoue, Yi-Jing Lee, Chung-Lin Jiang, Fu-Jung Lin

**Affiliations:** 1 Department of Animal Science, National Pingtung University of Science and Technology, 91201 Pingtung, Taiwan; 2 Howard Hughes Medical Institute, Harvard Medical School, 02115, Boston, USA; 3 Program in Cellular and Molecular Medicine, Boston Children’s Hospital, Boston, 02115 USA; 4 Department of Genetics, Harvard Medical School, Boston, MA 02115 USA; 5 Harvard Stem Cell Institute, Harvard Medical School, Boston, MA 02115 USA; 6 Institute of Molecular and Cellular Biology, National Taiwan University, Taipei, 10617 Taiwan; 7 Department of Biochemical Science and Technology, National Taiwan University, Taipei, 10617 Taiwan; 8 Research Center for Development Biology and Regenerative Medicine, National Taiwan University, Taipei, 10617 Taiwan; 9 Present Address: RIKEN Center for Integrative Medical Sciences, Yokohama, Kanagawa 230-0045 Japan

**Keywords:** Developmental biology, Germline development

## Abstract

Elongator complexes are well known to be involved in a wide variety of cellular processes; however, their functions in mammalian oocytes have not been characterized. Here, we demonstrated in mice that specific deletion of one of the core subunits, *Ikbkap/Elp1*, in oocytes resulted in spindle defects and chromosome disorganization without affecting folliculogenesis. In accordance with these findings, we observed that *Ikbkap* mutant female mice are subfertile. Further analyses uncovered that kinetochore–microtubule attachments are severely compromised in *Ikbkap*-deficient oocytes. Moreover, we revealed that *Ikbkap* modulates the acetylation status of α-tubulin in oocytes, which may at least in part mediate the meiotic phenotypes described above by affecting microtubule dynamics and kinetochore function. Finally, we showed that embryos derived from *Ikbkap*-deficient oocytes exhibit an increased frequency of aneuploidy, digyny, progressive delays in preimplantation development, and severe degeneration before reaching the blastocyst stage. In summary, we identify *Ikbkap* as an important player in regulating oocyte meiosis by modulating tubulin acetylation for chromosome/spindle organization.

## Introduction

Poor oocyte quality is a leading cause of female infertility. In mammals, primary oocytes are arrested at the prophase stage of the first meiotic division (MI), which is referred to as the germinal vesicle (GV) stage. Upon the stimulation of sex steroids secreted by the surrounding granulosa cells, GV oocytes reinitiate meiosis, as indicated by GV breakdown (GVBD). Then the oocytes progress through the metaphase (M)-phase of MI. Unlike spermatogenic meiosis, oogenic cytokinesis occurs unevenly (asymmetrical cytokinesis) to produce two daughter cells of different sizes. Metaphase spindle migrates to the periphery of the cell, which is followed by extrusion of the first polar body. Meanwhile, the oocyte immediately enters the M-phase of the second meiotic division (MII) to complete meiotic maturation. The egg is ovulated and remains arrested at the metaphase of meiosis II until fertilization occurs. Upon sperm penetration, the oocyte is activated and released from the MII arrest, resulting in completion of the second meiotic division. A similar asymmetric cytokinesis takes place during the second division of meiosis to extrude the second polar body, and the fertilized egg enters into the interphase of the first embryonic cell cycle. During oogenic meiosis, accurate regulation of spindle assembly and chromosome organization is essential for producing a healthy oocyte. Aberrant chromosome segregation during female meiosis could result in aneuploidy in the egg, which is the major cause of spontaneous miscarriages, trisomic conceptions, and elevated rates of infertility^1^. Although numerous molecular players and pathways have been shown to regulate spindle and chromosome organization in oocyte meiosis, the molecular mechanisms underlying chromosome segregation remain to be uncovered.

Elongator is a multi-subunit complex that plays pivotal roles in regulating multiple biological processes in response to a broad spectrum of cellular stimuli. Composed of two copies of a core complex (Elp1-3) and a sub-complex (Elp4-6)^2^, Elongator was first identified by its association with hyperphosphorylated RNA polymerase II in budding yeast^3^. Subsequent identification of Elongator in humans and other species revealed its evolutionary conservation from yeast to humans^4–6^. Besides its role in transcription, Elongator is also involved in translation, for it regulates tRNA modification. Elp1, also known as IKAP (Inhibitor of kappaB kinase-associated protein), serves as a scaffold protein required for the assembly of Elongator, and it is encoded by the *Ikbkap* gene. The catalytic component Elp3 contains a motif found in the GCN5-related N-acetyltransferase (GNAT) family of histone acetyltransferases (HATs)^7^. Of the acetylation targets, Elp3 has been shown to catalyze acetylation of histone H3 in the nucleus, Bruchpilot at the neuromuscular junction in flies, ɑ-tubulin in post-mitotic neurons, and connexin-43 in the cerebral cortex^8–11^. The functional importance of all subunits *in vivo* has been demonstrated, for deletion of any of the subunits leads to similar pleiotropic phenotypes in *Saccharomyces cerevisiae*, *Caenorhabditis elegans*, *Drosophila melanogaster* and *Arabidopsis thaliana*^12–17^.

We previously showed that ablation of *Ikbkap* in male germ cells affects spermatogenesis^18^. However, its role during oocyte meiosis remains unknown. Here, we uncover an essential role for *Ikbkap* in regulating mouse oogenesis. By disrupting *Ikbkap* specifically in female germ cells, we found that oocytes lacking *Ikbkap* were unable to complete meiosis and displayed defects in meiotic spindle organization, chromosome congression, and kinetochore function. In addition, an increase in aneuploidy rate was observed in *Ikbkap-*deficient oocytes. Moreover, we showed that zygotes lacking maternal *Ikbkap* exhibited a high incidence of digyny and abnormal preimplantation development. We discovered that IKAP plays an important role in governing spindle assembly and in chromosome organization and segregation during female meiosis by regulating tubulin acetylation.

## Results

### *Ikbkap* is expressed in growing oocytes and preimplantation mouse embryos

**Figure 1:**
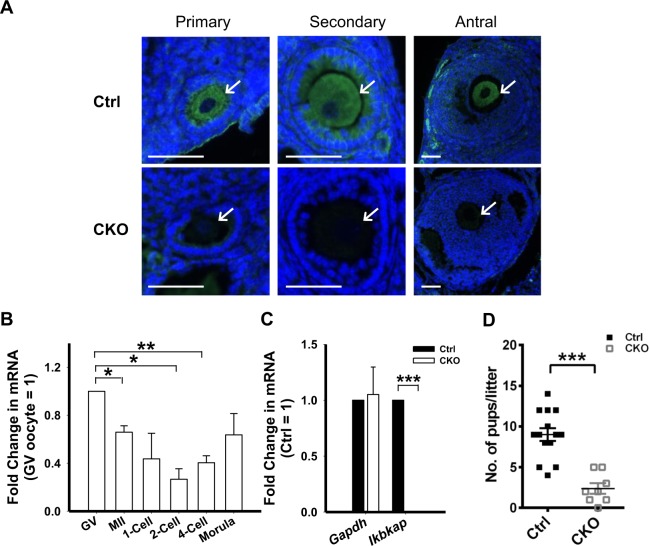
Expression pattern of *Ikbkap* during oogenesis and impact of deletion on fertility. (**A**) Immunofluorescence staining showing the levels of IKAP in the oocytes of primary, secondary and antral follicles. Ovaries from control (Ctrl) and *Ikbkap* mutant (CKO) mice were stained with anti-IKAP antibody. The sections were counterstained with DAPI to visualize DNA. Oocytes are indicated by arrows. Scale bar, 50 μm. (**B**) Graph shows the fold induction of *Ikbkap* mRNA levels in unfertilized GV, MII oocytes, and various stages of embryos compared with the values of GV oocytes. Quantitative reverse transcriptase-polymerase chain reaction (qRT-PCR) was performed. *Gapdh* was used as an internal control. (**C**) *Gapdh* and *Ikbkap* mRNA levels in Ctrl and CKO MII oocytes. (**D**) Fertility analysis of Ctrl and CKO female mice. Females were crossed with wild-type males. Litter size is shown as the number of pups per litter. Horizontal lines are the mean. Data are presented as mean ± standard error of the mean (SEM). *p < 0.05; **p < 0.01; ***p < 0.001.

To probe potential roles for *Ikbkap* in the mammalian female germline, we first examined its expression pattern during oocyte development. Immunofluorescence staining of paraffin-embedded sections of ovaries revealed that IKAP was highly expressed in the cytoplasm of the oocytes of primary, secondary and antral follicles (Fig. 1A), suggesting a potential role for *Ikbkap* during meiotic maturation. We further examined its expression levels in growing oocytes and early embryos using real-time PCR. Consistent with the staining results, *Ikbkap* mRNA appeared most intense in fully-grown germinal vesicle (GV) oocytes. We also observed modest expression of *Ikbkap* mRNA throughout the preimplantation stage, with a slight down-regulation at the 2-cell stage, suggesting that it plays a role during preimplantation development. To explore its functional relevance in the female germline, we disrupted *Ikbkap* in oocytes by combining *Ikbkap* floxed allele (*Ikbkap*^*flox*/*flox*^) with a *Cre* recombinase transgene expressed from the *Vasa* promoter (also known as *Ddx4-Cre*)^19^. The *Vasa-*promoter begins to drive transcription in primordial germ cells starting from embryonic day (E) 15.5 and in postnatal stages^19^. *Vasa-Cre* activity results in an oocyte-specific null allele through deletion of the exon 3 of *Ikbkap* in mice^18^, generating germ lineage-specific *Ikbkap* mutant mice (genotyped as *Vasa-Cre; Ikbkap*^*flox*/−^, referred to as CKO hereafter). Mice of other genotypes (*Vasa-Cre; Ikbkap*^*flox*/+^, *Ikbkap*^*flox*/+^, and *Ikbkap*^*flox*/*flox*^) produced from the breeding scheme exhibited no overt phenotypes and were used as the control group. Validation of the CKO mouse line was carried out using immunofluorescence staining and real-time PCR analysis. Immunofluorescence staining confirmed the significant reduction of IKAP proteins in oocytes (Fig. 1A). *Ikbkap* mRNA expression was almost completely abolished in growing CKO oocytes (Ctrl: 1; CKO: 0.004), indicating successful disruption of *Ikbkap* in oocytes (Fig. 1B).

### Female mice with oocyte-specific deletion of *Ikbkap* are subfertile

**Figure 2:**
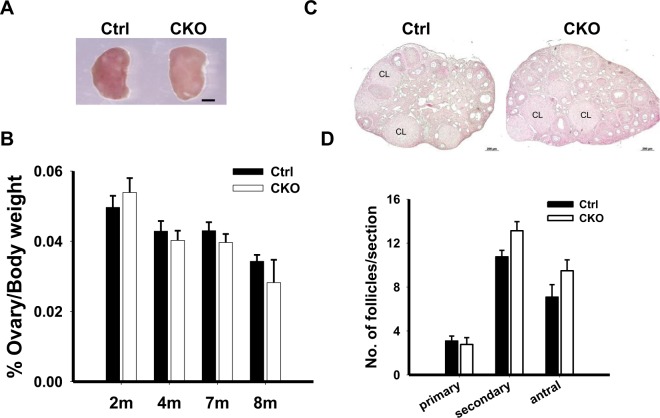
Folliculogenesis occurs normally in *Ikbkap* mutant female mice. (**A**) The images of the ovaries from 4-month-old Ctrl and CKO mice were captured using a light microscope. Scale bars, 1 mm. (**B**) Ovary weight at 2, 4, 7, and 8-month old Ctrl and CKO mice (n = 4–7). (**C**) Hematoxylin and Eosin (H&E) staining of ovaries from 4-month-old females. The overall development of follicles and corpora lutea (CL) in CKO mice was found to be unaffected compared to Ctrl mice. Scale bar, 200 μm. (**D**) Quantification of the different types of follicles per ovarian section. At least five sections per ovary from at least three mice per genotype were analyzed. Data are presented as the mean ± SEM.

To determine whether the loss of *Ikbkap* in oocytes affects fertility in female mice, a breeding assay was carried out by crossing control and CKO females with wild-type male mice of proven fertility. CKO females gave birth to markedly smaller-sized litters with an average of 2.4 pups, whereas their littermate control siblings had an average litter size of 9 pups (Fig. 1D), indicating that CKO females are subfertile. Nevertheless, we found that CKO ovaries were morphologically and histologically indistinguishable from those of controls (Fig. 2A,C). The ovary weight of sexually mature *Ikbkap* conditional null mutants at various ages (2, 4, 7 and 8 months old) was comparable to that of their littermate controls (Fig. 2B). Healthy corpora lutea (CL) along with follicles at various developmental stages ranging from primary to antral stages were identified in CKO ovaries at 16 weeks of age (Fig. 2C). In addition, the average numbers of follicles containing identifiable growing and fully-grown oocytes were similar between control and CKO ovaries (Fig. 2D). These data indicated that, although depletion of *Ikbkap* in oocytes leads to subfertility, *Ikbkap* appears to be dispensable for oocyte growth and follicle development.

### *Ikbkap* deficiency disrupts meiotic maturation

**Figure 3:**
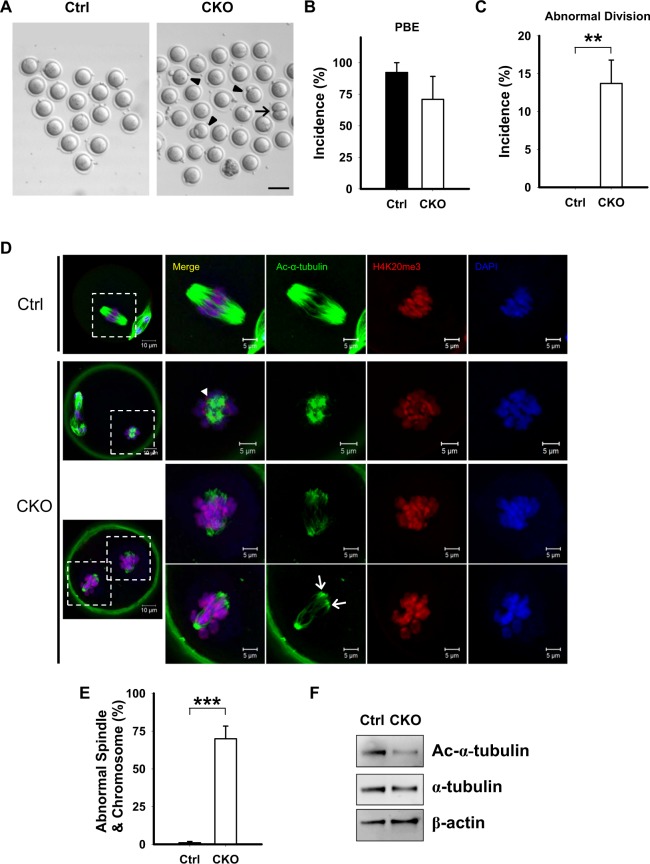
*Ikbkap* deficiency causes defects in oocyte maturation, spindle organization and chromosome alignment. (**A**–**C**) Fully-grown oocytes isolated from Ctrl and CKO mice were cultured for 16 h to evaluate the rate of polar body extrusion (PBE) (**B**) and abnormal division rate (**C**). (**A**) Photomicrographs of representative Ctrl and CKO oocytes. Arrowheads indicate oocytes that extruded a large-sized polar body; the arrow indicates an oocyte with apparent symmetrical division. Scale bar, 100 μm. (**B**,**C**) Quantitative analysis of Pb1 extrusion rate (**B**) and abnormal division rate (**C**) in Ctrl (*n* = 91) and CKO (*n* = 76) oocytes. Data are presented as mean ± SEM of three independent experiments. **p < 0.01 *vs*. Ctrl. (**D**) Ctrl and CKO oocytes were stained with acetylated α-tubulin antibody, H4K20me3 antibody, and DAPI to visualize the spindle (green), pericentric chromosomal regions (red), and chromosomes (blue), respectively. Ctrl MII oocytes presented a typical barrel-shaped spindle and well-aligned chromosomes on the metaphase plate. Apolar spindles, multipolar spindles (arrows) and chromosome misalignment were frequently observed in CKO MII oocytes. Scale bars, 5 µm. (**E**) Quantification of Ctrl and CKO oocytes with abnormal spindle or chromosome organization. Graph shows mean ± SEM of results obtained from at least six independent experiments in which ≥190 oocytes were analyzed. ***p < 0.001. (**F**) Western blot analysis showing the decreased acetylation of α-tubulin in CKO oocytes compared to the Ctrl group. The level of α-tubulin was not changed significantly in CKO oocytes compared to the Ctrl oocytes. β-actin served as a loading control.

To understand the cause of subfertility, we analyzed meiotic maturation in CKO oocytes. GV stage oocytes were obtained from CKO and their littermate controls and induced to undergo meiotic maturation *in vitro*. The incidence of polar body (PB) extrusion, which is indicative of the metaphase-to-anaphase transition of meiosis I, was determined. As shown in Fig. 3A and B, a considerable fraction of the CKO oocytes was able to develop to the MII stage, which was identified by the extrusion of a polar body (Ctrl: 92.2 ± 13.5%; CKO: 70.9 ± 31.5%; *p* > 0.05; Fig. 3A,B). Strikingly, 13.7% of the CKO oocytes exhibited abnormal division (Fig. 3C). Among them, a high number of CKO oocytes underwent a symmetric “2-cell-like” meiotic division (Fig. 3A, arrow) or had abnormal PB size (Fig. 3A, arrowheads). Taken together, these data suggest that *Ikbkap* is required for oocyte maturation and meiotic divisions.

### Deletion of *Ikbkap* impairs spindle organization and chromosome alignment

To determine whether *Ikbkap* ablation in oocytes affects the meiotic spindle, control and CKO oocytes were immunostained with anti-acetyl-α-tubulin and anti-H4K20me3 antibody to visualize the spindles and pericentric regions, respectively. Oocytes were counterstained with DAPI to label chromosomes. Confocal microscopy revealed that most control oocytes at the metaphase presented with a bipolar barrel-shaped spindle and well-aligned chromosomes on the metaphase plate (Fig. 3D). In contrast, a high frequency of spindle defects and chromosome disorganization was observed in CKO oocytes (69.9 ± 20.6%, *p* < 0.001; Fig. 3D,E). The phenotypes included apolar spindles (Fig. 3D, arrowhead), symmetric division, multipolar spindles (Fig. 3D, arrows), and severe chromosome misalignment (Fig. 3D). These results suggest that most *Ikbkap-*deficient oocytes cannot properly organize the meiotic spindle and/or align the meiotic chromosomes.

We also performed the staining on oocytes matured to the prometaphase (ProM) of MI. In control oocytes, the spindle assumed a typical bipolar organization, and chromosomes were positioned around each pole. Bipolar spindles nucleated highly developed radial arrays of astral microtubules, many of which extended to the cell cortex (Fig. S1A). In CKO oocytes, we observed catastrophic spindle disorganization defects similar to those observed in MII oocytes, including apolar spindles and short astral microtubules between the two poles (Fig. S1A). The incidence of abnormal spindle formation in the ProMI/ MI transition was significantly higher in CKO oocytes than in control oocytes (Fig. S1B). Together, these results suggest that *Ikbkap* depletion causes spindle defects and chromosome misalignment during meiosis of mouse oocytes.

### Acetylated tubulin is downregulated in *Ikbkap*-deficient oocytes

The multi-subunit Elongator complex harbors histone acetyltransferase activity. In addition to histones, IKAP/Elp1 has been shown to interact with and acetylate α-tubulin in neuron cells, which is crucial for the migration and differentiation of cortical neurons^9^. Tubulin is one of the most abundant non-histone proteins in oocytes that are subjected to acetylation, and acetylated tubulin was associated with stable microtubules at the spindle poles^20^. We then investigated whether *Ikbkap* deficiency would impact α-tubulin acetylation using immunofluorescent staining and Western blot analysis with anti-acetylated α-tubulin antibodies. The acetylation levels of α-tubulin were markedly decreased in CKO oocytes compared with those in control oocytes (Figs. 3D,F and S2). Western blot analysis and immunofluorescent staining revealed that α-tubulin levels were comparable between the two groups (Figs. 3F, S2 and S3). Taken together, the results indicate that the disruption of *Ikbkap* induced hypoacetylation of α-tubulin in mouse oocytes can result in spindle disorganization and chromosome mis-segregation by interfering with the microtubule network and kinetochore function.

### *Ikbkap* deficiency impairs kinetochore–microtubule interaction and induces aneuploidy in mouse oocytes

**Figure 4:**
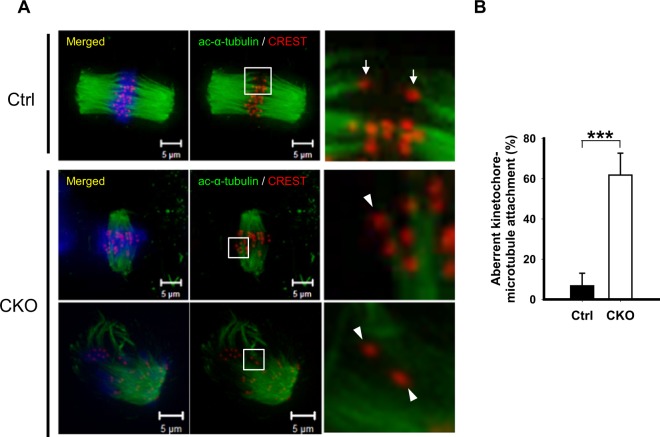
*Ikbkap-*deficient oocytes display impaired microtubule**–**kinetochore attachment. (**A**) Ctrl and CKO MII oocytes were immunostained for acetylated α-tubulin (green), CREST (red), and DAPI (blue). Representative confocal images are shown. Right panels show enlarged view of boxed areas in middle panels. Arrows indicate bioriented and attached kinetochores; arrowheads show defective and unattached kinetochores. Scale bars, 5 µm. (**B**) Quantification of Ctrl and CKO oocytes with impaired microtubule-kinetochore attachment. Data are expressed as mean ± SEM of results obtained from at least 3 independent experiments in which ≥45 oocytes for each group were analyzed. ***p < 0.001.

Accurate chromosome alignment and segregation requires the formation of spindle microtubules and kinetochores. Kinetochores are protein complexes that assemble on centromeres where the spindle attaches to and pull the chromosomes apart during cell division^21^. Given that the CKO oocytes exhibited spindle and chromosome disorganization, we investigated whether the kinetochore**–**microtubule (KT-MT) attachments were defective. Superovulated metaphase-II (MII) oocytes were immunostained with CREST anti-sera and acetylated α-tubulin antibodies to label kinetochores and microtubules, respectively. Chromosomes were co-stained with DAPI. Confocal microscopy revealed that the control oocytes formed stable kinetochore**–**microtubule attachments with each kinetochore attached to one pole, whereas CKO oocytes had a higher frequency of kinetochore**–**microtubule mis-attachments, such as unattached kinetochores, than did control oocytes (Fig. 4A). Of note, quantitative analysis revealed that aberrant kinetochore**–**microtubule attachment was significantly increased in *Ikbkap-*depleted oocytes (61.8 ± 10.9%, *p* < 0.001; Fig. 4A,B). These findings indicate that reduction in the pulling forces across kinetochores and the decreased stability of kinetochore microtubules in CKO oocytes could, at least in part, cause the chromosome alignment defects.

Misalignment of chromosomes generally leads to aneuploidy. To explore this possibility, we analyzed the karyotype of MII oocytes by chromosome spreading combined with kinetochore labeling. We showed that deletion of *Ikbkap* resulted in oocytes with abnormal numbers of chromosomes and kinetochores, which are characteristic of aneuploidy (Fig. S4A). The incidence of aneuploidy was significantly higher in CKO oocytes than in control oocytes (51.5 ± 4.8% in CKO vs. 10.3 ± 0.3% in Ctrl, *p* < 0.05; Fig. S4B). These observations suggest that the loss of *Ikbkap* impairs spindle assembly and chromosome segregation, consequently leading to the induction of aneuploidy.

### *Ikbkap* deficiency disrupts early embryonic development

**Figure 5:**
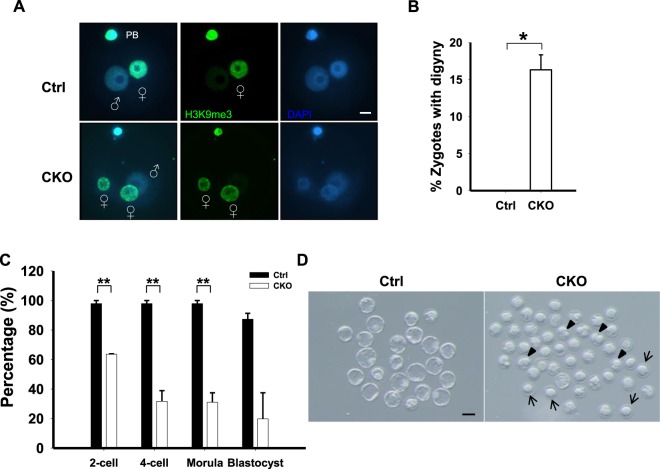
Embryos lacking maternal *Ikbkap* exhibited progressive developmental delays and failed to develop into blastocysts. (**A**,**B**) MII oocytes were fertilized *in vitro*, and zygotes at the pronuclear stage were collected. Zygotes were immunolabeled with H3K9me3 antibody to visualize female pronuclei (green), and chromosomes were counterstained with DAPI (blue). Shown are representative confocal images (**A**) and incidence of digynic zygotes from *in vitro* fertilized Ctrl (*n* = 52) and CKO (*n* = 144) oocytes. (**B**) *n* indicates the number of oocytes analyzed from at least two independent experiments. At least three Ctrl and three CKO females were used for each experiment. (**C**,**D**) MII oocytes isolated from superovulated Ctrl and CKO females were fertilized *in vitro*, and the embryos were cultured *in vitro* until the blastocyst stage. Their developmental stages were determined by morphology. Shown are the percentages of embryos at different stages (**C**) and the representative images of embryos at 120 hours post-fertilization (**D**). Abnormal embryos included those that were at the 1-cell stage (arrows) or 2-cell stage (arrowheads) or those exhibiting abnormal morphology. 47 Ctrl and 121 CKO embryos were examined at each time point. Data are presented as mean ± SEM of two independent experiments. Three mice per group were used for each experiment. *p < 0.05; **p < 0.01.

To assess the developmental competence of these oocytes, MII oocytes isolated from superovulated CKO females were fertilized *in vitro*. Zygotic development during the pronuclear stage was first examined. Strikingly, a portion of the CKO zygotes displayed three pronuclei (Ctrl: 0%; CKO: 16.3 ± 2.9%, *p* < 0.05; Fig. 5A,B). To distinguish between the parental pronuclei, we labeled female pronuclei and polar bodies with anti-H3K9me3 antibody and found that the extra-pronuclei were of maternal origin (Fig. 5A). The process of a diploid ovum fertilized by a haploid sperm is called digyny and results in triploidy^22^. These data indicated that tripronuclear (3PN) zygotes generated by CKO oocytes are digynic in origin, possibly due to failure in releasing the second polar body upon fertilization.

We also monitored early embryonic development until the blastocyst stage. After fertilization, in contrast to 97.9% of the control embryos, only 63.6% of the mutant embryos reached the two-cell stage (*p* < 0.001; Fig. 5C). Strikingly, only 19.8% of mutant one-cell zygotes developed into blastocysts, versus 87.3% of the one-cell stage zygotes from control oocytes (*p* < 0.001; Figs. 5C,D). At 120 hrs after fertilization, control embryos were predominately at the blastocyst stage with an obvious blastocoel, whereas most of the mutant embryos had barely reached the blastocyst stage (Fig. 5D). A high percentage of mutant embryos were at the 1-cell stage (Fig. 5D, arrows) or 2-cell stage (Fig. 5D, arrowheads). These data revealed that, although CKO MII oocytes were fertilizable, embryos lacking maternal *Ikbkap* showed progressive delays in development, with more than 60% of them undergoing degeneration prior to the four-cell stage. These observations suggest that defective preimplantation development derived from *Ikbkap* mutant zygotes may account for the subfertility observed in CKO mice.

## Discussion

The current study investigated the functions of *Ikbkap* during oogenesis and female meiosis. We showed that *Ikbkap-*depleted oocytes showed a full spectrum of abnormal phenotypes, including a disorganized spindle, improper chromosome alignment, impaired microtubule**–**kinetochore interaction, an increased aneuploidy rate, a high incidence of digyny, and abnormal preimplantation development, which contributed to subfertility in the *Ikbkap-*deficient females. Moreover, we showed that depletion of *Ikbkap* impaired the acetylation of α-tubulin, suggesting that Elongator promotes α-tubulin acetylation in oocytes, which is critical for the maintenance of spindle morphology and kinetochore function.

Microtubules, which are formed by α- and β-tubulin heterodimeric complexes, are required for a variety of fundamental cellular processes, including cell motility, mitosis, movement of organelles and vesicles through the cytoplasm, establishment of cell polarity and maintenance of cell morphology^23^. The functions and properties of microtubules are controlled by post-translational modifications of their tubulin subunits. For example, α-tubulin can be modified by phosphorylation, tyrosination, detyrosination, glycylation, glutamylation and acetylation^24^. Among them, acetylation of α-tubulin on lysine 40 is a common modification that occurs in oocytes, but its functions during meiosis are largely unknown. Acetylated α-tubulin is enriched at the spindle poles of mouse oocytes, and its signal is weaker throughout the spindle^20^. In mammalian cells, acetylated microtubules commonly resist drug-induced disassembly and mechanical bending-induced damage^25,26^. They also regulate the activity of microtubule-associated proteins and control motor-protein trafficking^27,28^. These findings suggest that tubulin acetylation is a marker of long-lived stabilized microtubules^29^. One could speculate that α-tubulin hypoacetylation decreases microtubule stability and/or kinetochore**–**microtubule interaction, which leads to defective meiotic spindles, misaligned chromosomes during oocyte meiosis, and reduced oocyte quality. Indeed, we observed these effects in *Ikbkap-*deficient oocytes. Chromosome misalignment and disruption of chromosome movement during meiosis could result in aneuploidy, which is a leading genetic cause of infertility^1^. These could be the mechanisms underlying reduced fertility in *Ikbkap* knockout female mice. In line with this notion, recent studies revealed that tubulin acetylation is essential for the maintenance of spindle organization and chromosome segregation in histone deacetylase (HDAC) 3- or 6-deficient oocytes^30,31^. Notably, we could not exclude the possibility that the spindle disorganization phenotype in *Ikbkap-*depleted oocytes might have been caused by acetylation of targets other than spindle α-tubulin.

Several other candidate acetyltransferases have been suggested to mediate α-tubulin acetylation in various mammalian cell types, including αTAT1^32^, arrest defective 1 (ARD1)-N-acetyltransferase 1 (NAT1) complex^33^, GCN5^34^, and ELP3^9^; however, their functions in female meiosis have not been reported. The reduced levels of, but not the lack of, lysine 40 α-tubulin acetylation in *Ikbkap-*deficient oocytes indicate that Elongator is unlikely to be the only α-tubulin acetyltransferase in oocytes. Furthermore, deletion of the α-tubulin deacetylases *Sirt2*, *Hdac3* and *Hadc6* in mouse oocytes resulted in hyperacetylation of α-tubulin, leading to meiotic spindle disorganization^30,31,35^. Collectively, our and others’ data indicate that fine-tuned regulation of α-tubulin acetylation is critical for female meiotic progression.

Our previous and current studies have indicated that *Ikbkap* is important for both male and female gametogenesis^18^. However, its ablation using *Vasa-Cre* activity causes minor gender-specific consequences. In males, mutant mice are infertile and mutant spermatocytes exhibit severe defects in prophase I, whereas in females, CKO mice are subfertile and mutant oocytes show impaired metaphase**–**anaphase transition. This sexual dimorphism could be due to the timing of male and female meiosis and the onset of deletion of *Ikbkap*. In male mice, meiosis starts after puberty and continues throughout life, while in females, the process begins between E13.5 and E15.5, and it does not resume until puberty. As *Vasa-Cre* activity is strongly induced between E15 and E18^19^, the late onset of *Vasa-Cre* expression could lead to late onset of phenotypes and/or incomplete elimination of *Ikbkap* in female oocytes. An alternative approach is to use zona pellucida 3 (*Zp3*)-*Cre* driver, which induces *Cre* recombinase specifically in the growing oocyte prior to the completion of the first meiotic division^36^. It would be interesting to study whether early onset of *Ikbkap* elimination in oocytes could lead to similar infertile phenotypes.

In conclusion, our results reveal a role for *Ikbkap* during oocyte maturation and preimplantation development. We showed that Elongator modulates female meiotic progression by promoting α-tubulin acetylation, which is crucial for the stability of the spindle. Disruption of spindle and kinetochore function leads to the generation of aneuploid eggs and developmental defects in embryos.

## Methods

### Mouse strains and genotyping

*Ikbkap* ^*flox/flox*^ mice and *Vasa- Cre* mice were published previously^18,19^. Experimental mice were maintained in a mixed genetic background (129/Sv x C57BL/6) and received standard rodent chow. The primer sequences used for genotyping were described previously^18^. Experimental animals and the studies were reviewed and approved by the Institutional Animal Care and User Committee (IACUC) of Harvard Medical School and National Taiwan University. All procedures complied with the “Guide for the Care and Use of Laboratory Animals”.

### Oocyte collection and maturation

Fully-grown GV oocytes were obtained from the ovaries of superovulated female mice 48 h after intraperitoneal injection of 7.5 IU of pregnant mare serum gonadotropin (PMSG) as described previously^37^. Ovaries were placed in a Petri dish with M2 medium (Sigma-Aldrich) supplemented with IBMX (Sigma-Aldrich) to prevent oocytes from undergoing GVBD. GV oocytes were released by puncturing antral follicles with a fine needle under a dissecting microscope. To collect MII-stage oocytes, 7.5 IU of human chorionic gonadotropin (hCG) was administered 48 h after PMSG injection to induce ovulation. Mice were sacrificed 15**–**16 h post hCG by cervical dislocation and cumulus-oocyte complexes (COCs) were released from the oviducts. COCs were briefly incubated in M2 medium containing 0.3 mg/mL hyaluronidase (Merck Millipore) to remove cumulus cells. For *in vitro* maturation, oocytes were washed and cultured in IBMX-free M16 medium (Sigma-Aldrich) for various periods of time at 37 °C in 5% CO_2_ atmosphere.

### Collection of sperm and *in vitro* fertilization (IVF)

IVF was performed according to a previously described method^37^. Sperm were collected from the caudal epididymes of adult BDF1 male mice. The spermatozoa were activated for insemination by incubation for 1 h in the Human Tubal Fluid (HTF) medium before they were used. Oocytes that had been transferred to the HTF medium supplemented with 10 mg/mL bovine serum albumin (BSA; Sigma-Aldrich) were inseminated with activated spermatozoa. 6 h after insemination, fertilized oocytes were washed and cultured in KSOM (Millipore) in a humidified atmosphere of 5% CO_2_/95% air at 37 °C. 1-cell, 2-cell, 4-cell, and 8**–**16-cell stage embryos were collected at 11, 30, 48, and 72 h post-insemination (hpi), respectively.

### Histological analysis

Ovaries were collected and fixed in 4% paraformaldehyde (PFA, Electron Microscopy Sciences) overnight, followed by processing and embedding in paraffin. Ovaries were serially sectioned at 7 μm and stained with hematoxylin and eosin (H&E).

### Immunofluorescence analysis

Immunofluorescence staining was performed according to a method described previously^37^. Oocytes were fixed in 2% formaldehyde and 0.2% Triton X-100 for 1 h at 37 °C and then permeabilized with PBS containing 0.3% BSA and 0.1% Triton X-100 for 40 min. After blocking in PBS containing 0.3% BSA for 1 h, oocytes were incubated with anti-acetylated alpha tubulin antibodies (Merck Millipore), anti-alpha tubulin antibodies (Sigma), anti-H4K20me3 antibodies (Merck Millipore), and CREST (Antibodies, Inc.) at 4 °C overnight. Zygotes were fixed in 3.7% PFA in PBS containing 0.2% Triton X-100 for 20 min and then washed with PBS containing 10 mg/mL BSA (PBS/BSA). After blocking in PBS/BSA overnight, the samples were incubated with anti-H3K9me3 antibodies (Merck Millipore) for 2 hr at room temperature. After washing with PBS/BSA for 1 hr, they were incubated with a 1:500 dilution of Alexa Flour 488 goat anti-mouse IgG, Alexa Flour 568 goat anti-rabbit IgG, and Alexa Flour 647 goat anti-human IgG (Thermo Fisher Scientific) at 4 °C overnight. Oocytes or zygotes were then mounted on a glass slide in Vectashield Antifade Mounting Medium with 4’,6-diamidino-2-phenylindole (DAPI) (Vector Laboratories). Fluorescence images were taken using a laser-scanning confocal microscope with a spinning disk (CSU-10, Yokogawa) and an EM-CCD camera (ImagEM, Hamamatsu) or Zeiss LSM 700 confocal microscope. All images were acquired and analyzed using Axiovision or ZEN software (Carl Zeiss).

### Quantitative RT-PCR analysis

Total RNA was isolated from mouse oocytes using Trizol (Thermo Fisher Scientific). Genomic DNA was removed from purified RNA samples using Turbo DNase (Thermo Fisher Scientific). cDNA synthesis was performed with a SuperScript III First Strand synthesis kit and random primers according to the manufacturer’s directions (Thermo Fisher Scientific). Quantitative RT-PCR analyses were carried out using the ViiA7 Real-Time PCR System (Thermo Fisher Scientific) and FastStart Universal SYBR Green Master (Roche Applied Science). The expression data were normalized to *Gapdh*. Genomic DNA contamination was monitored by comparing samples with and without the step of reverse transcription.

### Western blot analysis

A total of 56 oocytes were lysed in RIPA buffer (Thermo Fisher Scientific, USA) containing a protease inhibitor cocktail (Roche) and heated for 5 min at 100 °C. Total oocyte proteins were subjected to electrophoresis on a 4–12% ExpressPlus PAGE gel (GeneScript Biotech Corp) and blotted onto PVDF membranes (GE Healthcare life science). The membranes were blocked in 5% BSA (BioShop) and incubated with primary antibodies directed against IKAP (Abcam), α-tubulin (Sigma-Aldrich), acetyl-α-tubulin (Merck), and β–actin (GeneTex). After three washes in TBST, the blots were then incubated with anti-rabbit or anti-mouse IgG antibody conjugated to horseradish peroxidase for 1 h. Enhanced chemiluminescence was performed according to the manufacturer’s instructions (GE Healthcare life science).

### Statistical analysis

Statistical analysis was performed using the Student *t*-test. Data are expressed as mean ± standard error of the mean (SEM), and *p* < 0.05 was considered statistically significant.

## Supplementary information

Supplementary Information
