# Supplementary material for: Loss of Ikbkap/Elp1 in mouse oocytes causes spindle disorganization, developmental defects in preimplantation embryos and impaired female fertility
Source: Sci Rep. 2019 Dec 11;9:18875. doi: 10.1038/s41598-019-55090-1 (PMC6906334; doi:10.1038/s41598-019-55090-1)
Supplement: Supplementary file 1 — Supplementary Information [file 41598_2019_55090_MOESM1_ESM.pdf]

Loss of *Ikbkap/Elp1* in mouse oocytes causes spindle disorganization, developmental defects in preimplantation embryos and impaired female fertility

Kuo-Tai Yang, Azusa Inoue, Yi-Jing Lee, Chung-Lin Jiang, and Fu-Jung Lin*

*Correspondence should be addressed to: Fu-Jung Lin, fujlin@ntu.edu.tw

SUPPLEMENTAL MATERIAL

Supplemental method
Supplemental reference
Supplemental figures 1-4

SUPPLEMENTAL METHOD

Chromosome Spreads of Mouse Oocytes

Chromosome spreading was performed according to the previously described method[1]. Oocytes were treated with Tyrode's solution (Sigma-Aldrich) to remove the zona pollucida, and then washed with M2 medium. The oocytes were incubated in hypotonic solution containing 50% fetal bovine serum for 10 min at 37°C. The oocytes were then dispersed on a coverslip that contained the fixation solution (1% paraformaldehyde, 70 mM dithiothreitol, and 0.15% Triton X-100, pH 9.2), followed by air-drying of the coverslip for 2 h.

SUPPLEMENTAL REFERENCE

1. Yang, K., Li, S., Chang, C., Tang, C., Lin, Y., Lee, S. , Tang, T.. Aurora-C Kinase Deficiency Causes Cytokinesis Failure in Meiosis I and Production of Large Polyploid Oocytes in Mice. *Mol Biol Cell.* **21(14)**: 2371–2383 (2010).

Figure S1

A

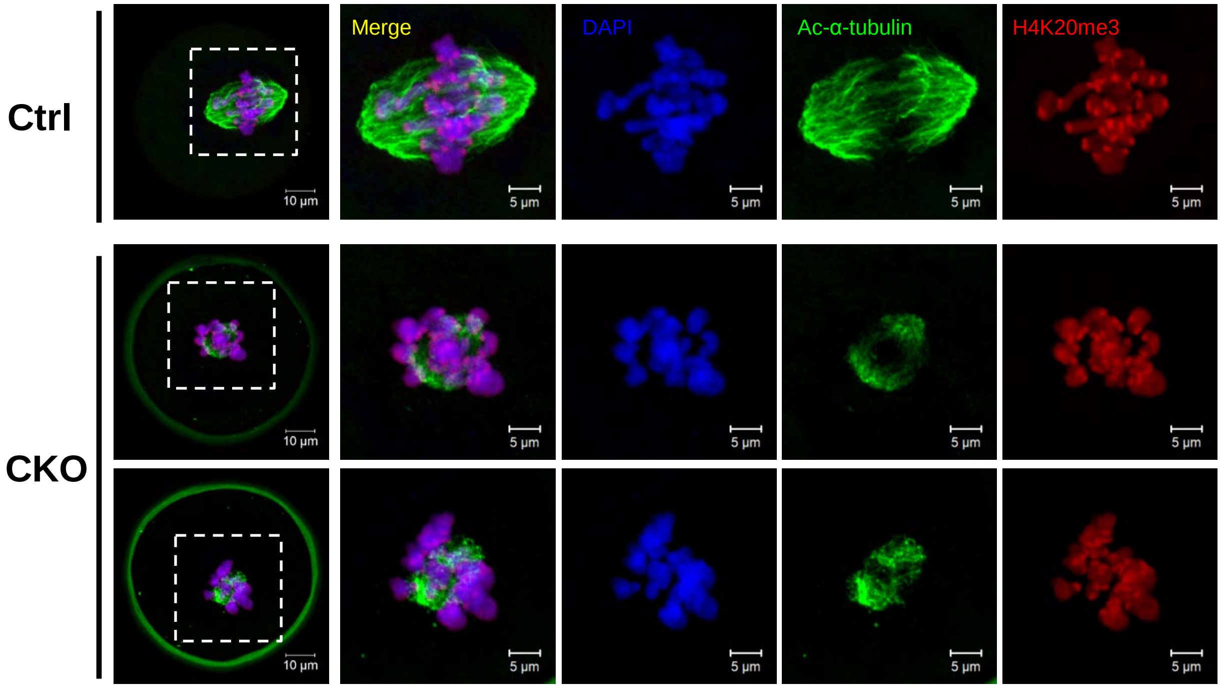

B

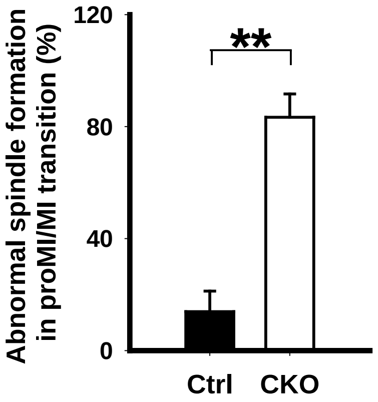

Figure S1. Loss of *Ikbkap* in oocytes causes defects in spindle organization and chromosome alignment during prometaphase I/metaphase I transition. (A, B) GV oocytes isolated from Ctrl and CKO mice were cultured *in vitro* to monitor prometaphase I/metaphase I transition . (A) Photomicrographs of representative Ctrl and CKO oocytes. Ctrl and CKO oocytes were stained with acetylated α-tubulin antibody (green), H4K20me3 antibody (red), and DAPI (blue). Scale bars, 5 μm. (B) Quantitative analysis of abnormal spindle formation rate in Ctrl (n = 14) and CKO (n = 13) oocytes. Data are presented as mean ± SEM of three independent experiments. $^{**}p < 0.01$ *vs*. Ctrl.

Figure S2

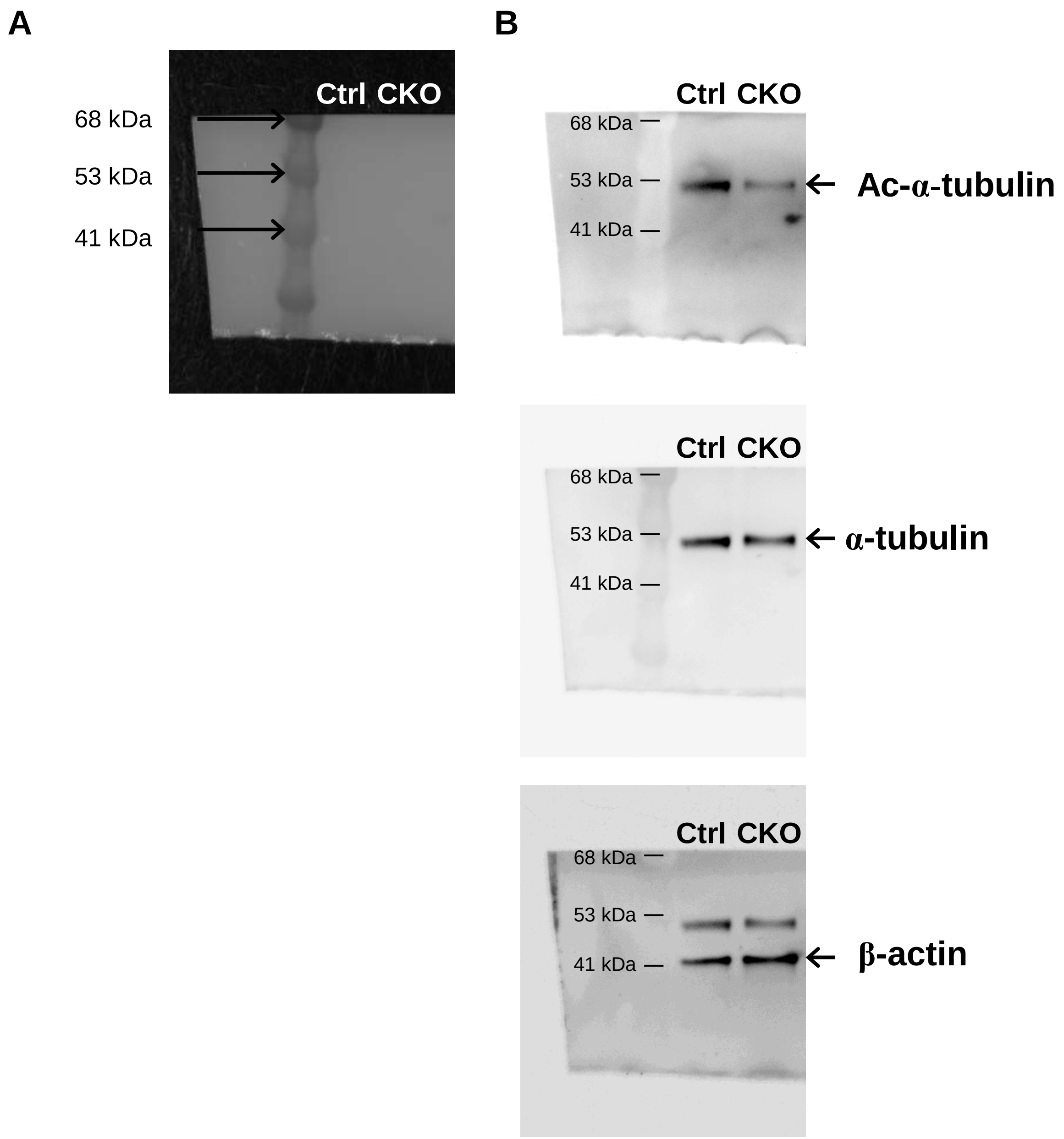

Figure S2. Full-length blots corresponding to Figure 3F. (A) The figure is showing full length original blot images. The size (kDa) of the protein markers are indicated on the left side of the figures. (B) Western blot analysis of acetylated α-tubulin (top). Blots were stripped and re-probed with α-tubulin (middle). Then blots were probed with β-actin (bottom).

Figure S3

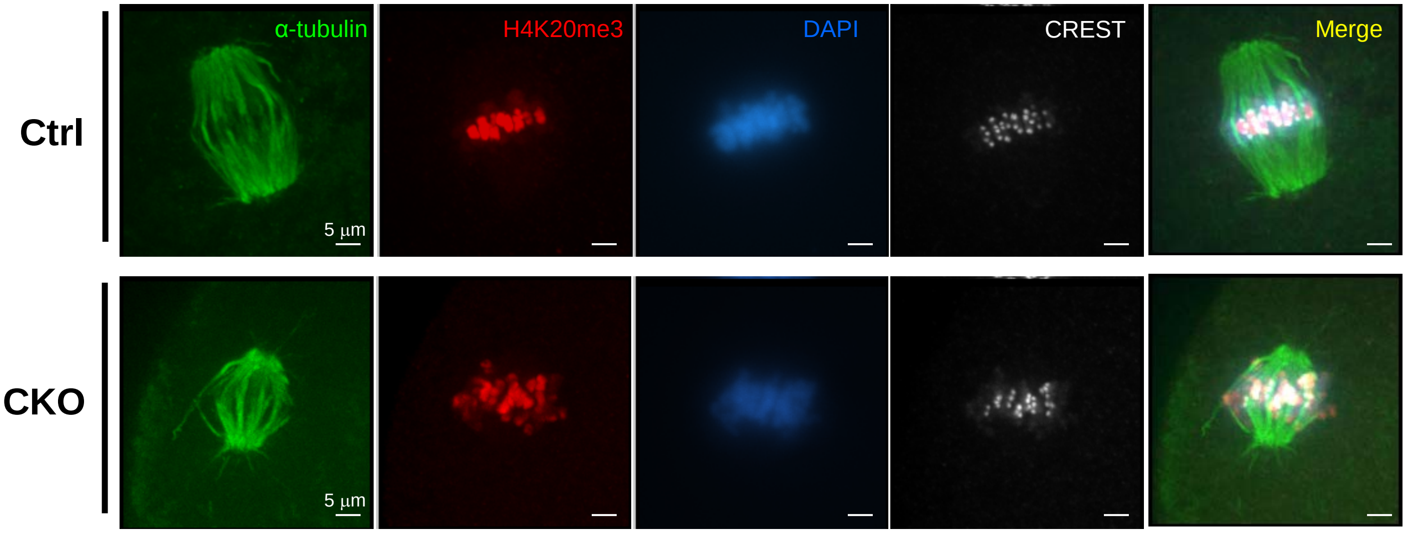

Figure S3. α-tubulin signal was unaffected in *Ikbkap* deficient oocytes. Photomicrographs of representative Ctrl and CKO oocytes. Ctrl and CKO MII oocytes were stained with α-tubulin antibody (green), H4K20me3 antibody (red), DAPI (blue), and CREST (white). Scale bars, 5 μm.

Figure S4

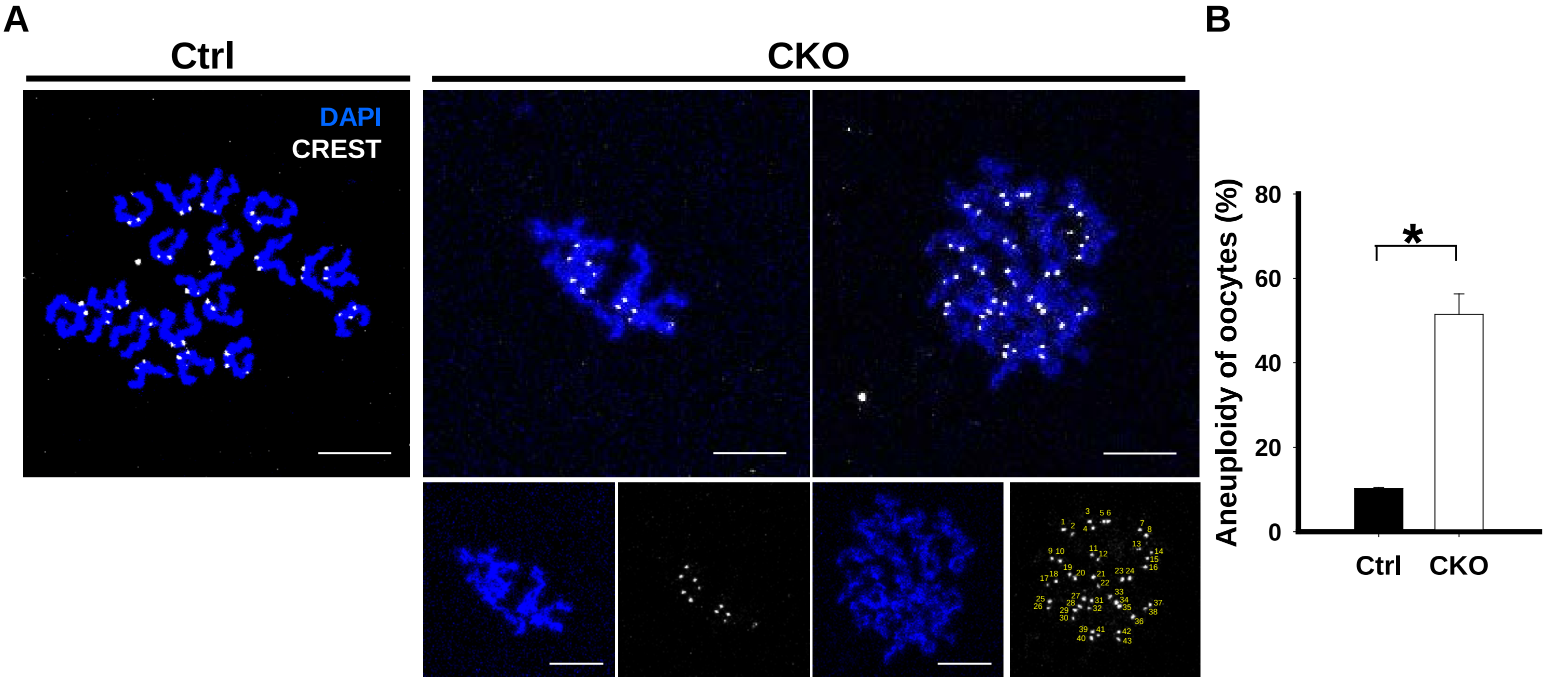

Figure S4. Loss of *Ikbkap* in oocytes results in increased incidence of aneuploidy. (A) Chromosome spread of control and *Ikbkap* depleted MII oocytes. Chromosomes were stained with DAPI (blue) and kinetochores were labeled with CREST (white). Representative confocal images of an euploid Ctrl oocyte (left), a hypohaploid CKO oocyte (middle), and a hyperhaploid CKO oocyte (right). *Ikbkap* depleted oocytes exhibited abnormal number of chromosomes and kinetochores. Scale bars, 10 µm. (B) Quantification of aneuploidy in Ctrl (n = 30) and CKO (n = 31) oocytes. Data were presented as mean ± SEM of two independent experiments. *$p < 0.05$ *vs*. Ctrl.
